# Measurement of 1,5-anhydroglucitol in blood and saliva: from non-targeted metabolomics to biochemical assay

**DOI:** 10.1186/s12967-016-0897-6

**Published:** 2016-05-18

**Authors:** Anna Halama, Michal Kulinski, Sara Abdul Kader, Noothan J. Satheesh, Abdul Badi Abou-Samra, Karsten Suhre, Ramzi M. Mohammad

**Affiliations:** Department of Physiology and Biophysics, Weill Cornell Medicine-Qatar, Qatar-Foundation, P.O Box: 24144, Doha, Qatar; Translational Research Institute, Academic Health System, Hamad Medical Corporation, PO Box 3050, Doha, Qatar; Department of Internal Medicine, Hamad Medical Corporation, Doha, Qatar; Institute of Bioinformatics and Systems Biology, Helmholtz Zentrum München, German Research Center for Environmental Health, Neuherberg, Germany

## Abstract

**Background:**

Diabetes testing using saliva, rather than blood and urine, could facilitate diabetes screening in public spaces. We previously identified 1,5-anhydro-d-glucitol (1,5-AG) in saliva as a diabetes biomarker. The Glycomark™ assay kit is FDA approved for 1,5-AG measurement in blood. Here we evaluated its applicability for 1,5-AG quantification in saliva.

**Methods:**

Using pooled saliva samples, we validated Glycomark™ assay use with a RX Daytona^+^ clinical chemistry analyser. We then used this set-up to analyse 82 paired blood and saliva samples from a diabetes case–control study, for which broad mass spectrometry-based characterization of the blood and saliva metabolome was also available. Osmolality was measured to account for potential variability in saliva samples.

**Results:**

The technical variability of the read-outs for the pooled saliva samples (CV = 2.05 %) was comparable to that obtained with manufacturer-provided blood surrogate quality controls (CV = 1.38–1.8 %). We found a high correlation between Glycomark assay and mass spectrometry measurements of serum 1,5-AG (r^2^ = 0.902), showing reproducibility of the non-targeted metabolomics results. The significant correlation between the osmolality measurements performed at two independent platforms with the time interval of 2 years (r^2^ = 0.887), also indicates the sample integrity. The assay read-out for saliva was not correlated with the mass spectrometry-based 1,5-AG saliva measurements. Comparison with the full saliva metabolome revealed a high correlation of the saliva assay read-outs with galactose.

**Conclusions:**

Glycomark™ assay read-outs for saliva were stable and replicable. However, the signal was dominated by galactose, which is biochemically similar to 1,5-AG and absent in blood. Adapting the 1,5-AG kit for saliva analysis will require enzymatic depletion of galactose. This should be feasible, since the assay already includes a similar step for glucose depletion from blood samples.

## Background

Diabetes incidence has dramatically increased in recent years [1], with estimates suggesting that diabetes will affect 7.7 % of all adults (439 million) worldwide by 2030 [2]. This epidemic is particularly impacting countries in the Gulf region—including Qatar, the United Arabic Emirates, and Saudi Arabia—where diabetes affects around 20 % of the adult population, and there is a relatively high rate of undiagnosed diabetes, including among young adults and children [3, 4]. Uncontrolled diabetes, manifested by periods of hypo- or hyper-glycaemia, can lead to the development of chronic comorbidities of diabetes, including cardiovascular disease, retinopathies, and nephropathies [5]. Early diagnosis enables tight glycaemic control, and can improve patient outcomes by delaying or avoiding chronic complications. Most diabetes-related medical costs are associated with chronic complications and comorbidities [6]. Therefore, screening, monitoring, and maintaining blood glucose levels within the targeted range are fundamental for both patients and their health systems.

The blood level of haemoglobin A1c (HbA1C) is a clinically well-established marker of long-term glycaemic control [7]. The haemoglobin glycosylation rate depends on the blood glucose concentration, and therefore reflects long-term glycaemic control associated with erythrocyte turnover [8]. The American Diabetes Association recommends HbA1C monitoring as a standard protocol for patients with diabetes, along with self-monitoring of blood glucose levels [9]. A commonly used marker of short-term glycaemic control is 1,5-anhydro-D-glucitol (1,5-AG), a metabolically stable small molecule (metabolite) that is well absorbed in the intestinal tract and mainly originates from the diet [10]. In healthy individuals, 1,5-AG blood level remains relatively constant due to equilibrium between 1,5-AG absorption, urinary reabsorption, and secretion by the intestinal tract [10], with little or no biochemical transformation in the body. In hyperglycaemia (above the renal threshold of 180 mg/mL), this equilibrium is upset by increased 1,5-AG secretion caused by competitive inhibition of urinary reabsorption by glucose. Based on this process, biochemical assays have been developed to monitor 1,5-AG as a marker of short-term glycaemic control [11].

Monitoring blood 1,5-AG levels to assess short-term glycaemic control was introduced into clinical practice in Japan over 20 years ago [12, 13], and was established worldwide in 1996 [14]. Today, automated and quantitative 1,5-AG measurement can be performed using the commercially available biochemical assay kits GlycomarkTM (GlycoMark, Inc., USA) [15, 16], and Determiner-L (Kyowa Medex, Japan) [11]. In 2003, the US FDA approved the use of the GlycomarkTM assay for monitoring 1,5-AG as a marker of short-term blood glucose levels [17], which has since been evaluated in several clinical studies [18–22]. The first US clinical trial of the GlycomarkTM assay demonstrated that 1,5-AG levels sensitively and rapidly reflected glycaemia changes following personalized treatment strategy modifications—including changes in medication types or dosage, and initiation of insulin therapy or combination of different insulin regimens [22]. Another clinical trial used 1,5-AG measurements to compare two distinct insulin regimens in 233 patients with T2D, showing that 1,5-AG levels significantly differentiated between patients receiving different treatments, consistent with the independently monitored glucose profiles [18]. The authors of that study suggested 1,5-AG level monitoring as a tool for selecting and optimizing therapy for T2D patients [18].

We recently employed mass spectrometry (MS) combined with non-targeted metabolomics to identify type 2 diabetes (T2D)-associated metabolites in saliva, blood, and plasma samples [23, 24]. In a cross-sectional case/control study including 188 cases and 181 controls, we searched for diabetes biomarkers in saliva or urine samples that could enable development of a non-invasive strategy for diabetes screening and monitoring. We found that 1,5-AG levels in both saliva and plasma samples significantly distinguished diabetic patients from healthy controls. Moreover, 1,5-AG levels in plasma were significantly correlated with 1,5-AG levels in blood [23]. Our results support the possibility of measuring saliva 1,5-AG level as a new non-invasive strategy to be used together with HbA1C for diabetes screening and assessment of glycaemic control.

Although our previous findings indicate that all clinically established properties of the 1,5-AG blood marker can be extrapolated to its saliva equivalent, there is presently no easy and scalable method for 1,5-AG measurement in saliva in a clinical setting. Given the established value of 1,5-AG monitoring in diabetes care, and the availability of a biochemical assay to measure 1,5-AG in blood, here we aimed to evaluate the applicability of the Glycomark™ assay for monitoring 1,5-AG in saliva.

We tested assay robustness and reproducibility by repetitive measurements of identical saliva samples in a single machine run as well as over a period of 5 days in replicates. For validation purposes, we measured with Glycomark™ assay 82 paired plasma and saliva samples, for which broad mass spectrometry-based characterization metabolome was also available together with osmolality measurements for saliva. The correlation between assay readouts and metabolite intensities was determined to rule out potential sample mix-up.

## Methods

### Study design

This study was a collaboration between the Translational Research Institute (TRI) of Hamad Medical Corporation (HMC) and Weill Cornell Medical College-Qatar (WCMC-Q). The study design included two phases. First, we evaluated the stability and reproducibility of 1,5-AG measurements using the Glycomark™ assay using kit-provided control samples (surrogate serum matrix spiked with 1,5-AG) and pooled saliva samples from healthy individuals. Second, we used the Glycomark™ assay to analyse 82 paired saliva and plasma samples that were previously analysed using a non-targeted metabolomics platform [23]. The 1,5-AG measurements were performed 2 years after initial metabolomics study. Independent measurement of this parameter was performed to account for the potential variability in saliva osmolality.

### Preparation of a saliva master mix

Saliva samples were collected from 24 healthy individuals using the Salivette^®^ system, following the manufacturer’s recommendations. Briefly, each donor was first asked to rinse their mouth with water. They were then asked to chew the Salivette roll for 1 min so that it could absorb their saliva. The chewed Salivette roll was placed into the Salivette^®^ tube, which was centrifuged at 2500*g* for 10 min at 4 °C. After centrifugation, 200 µL of saliva was transferred into a collection tube. To create a pooled sample, we also transferred 850 µL from each tube into a 50 mL collection tube. The saliva mix was vortexed and transferred in aliquots of 200 µL to 102 vials, which were frozen at −80 °C until analysis. In the following sections, these samples are referred to as master mix (MM). The individual samples in separate collection tubes were also frozen at −80 °C until analysis.

### Characteristics of individual samples evaluated by non-targeted metabolomics and biochemical assay

We used blood and saliva samples collected as part of the Qatar Metabolomics Study on Diabetes (QMDiab) study protocol, which was approved by the Institutional Review Boards of HMC and Weill Cornell Medical College-Qatar (WCMC-Q) (research protocol number 11131/11). All study participants gave their written informed consent and authorized utilization of their samples for future research. The samples were de-identified, and a certificate of non-human research was obtained from the WCMC-Q research compliance office.

The QMDiab study was a collaboration between the Dermatology Department of Hamad Medical Corporation (HMC) and Weill Cornell Medical College-Qatar (WCMC-Q), and has been previously described [23, 24]. The primary aim was to identify metabolic markers of diabetes in saliva, blood, and urine samples. Briefly, questionnaires were used to collect information regarding age, gender, ethnicity, and history of T2D. Non-fasting saliva, plasma, and urine samples were collected and processed following standardized protocols [23]. HbA1C levels were measured at the Department of Laboratory Medicine and Pathology of HMC (Cobas 6000; Roche Diagnostics). Metabolic profiling of plasma, saliva, and urine samples was performed using a non-targeted metabolomics platform (Metabolon). A total of 2178 metabolites were analysed in 1043 samples [23].

For our present study, we retrieved 82 de-identified samples: 49 from healthy controls and 28 from patients with diabetes, selected based on the information regarding the history of T2D from questionnaires. This sample subset was selected based on the availability of surplus saliva aliquots. Table 1 presents the HbA1C values and age and gender data for this study group.

**Table 1:** Sample characteristics

Subject	Control (*n* = 49)	Type 2 diabetes (*n* = 28)	All (*n* = 82)^a^
Age, years	46.7 (26–71)	42.4 (24–67)	47.2 (24–71)
Female sex	26 (53 %)	15 (54 %)	44 (54 %)
HbA1C, µg/mL	6.3 (4.7–9.4)	6.5 (4.7–12.4)	6.5 (4.7–12.4)

Age and HbA1C values are shown as median (range); female sex is shown as number of subjects (percentage)

^a^All subjects, including 49 control subjects, 28 patients with type 2 diabetes, and 5 subjects for whom no information was available regarding health condition

### Metabolomics measurements

Metabolite profiling was performed at Metabolon Inc., using ultra-high-performance liquid chromatography-mass spectrometry (UPLC-MS) and gas chromatography-mass spectrometry (GC–MS) as previously described [25]. Briefly, saliva and plasma samples were extracted using a series of organic and aqueous solvents to remove the protein fraction. To analyse samples using both the UPLC-MS and GC–MS approaches, each extracted sample was divided into two equal parts. The organic solvent was removed and the samples were frozen and vacuum-dried.

The samples for UPLC-MS analysis were reconstituted in an acidic or basic solution compatible with LC-solvents. Extracts reconstituted in acidic conditions were measurements in acidic positive ion, optimized conditions and extracts reconstituted in basic conditions were measured in basic negative ion, optimized conditions. Measurements were performed on Waters ACQUITY UPLC system and a Thermo-Finnigan LTQ mass spectrometer equipped with an electrospray ionization (ESI) source and a linear ion-trap (LIT) mass analyser [25].

The GC–MS samples were derivatised prior to analysis. Sample separation was performed on a 5 % diphenyl/95 % dimethyl polysiloxane GC column, with helium as the carrier gas, and the temperature ramping up from 60° to 340 °C over 17.5 min. Measurements were performed on a Thermo-Finnigan Trace DSQ fast-scanning single-quadruple mass spectrometer using electron impact ionization [24].

Peaks were identified using Metabolon’s propriety peak integration software, which compared the obtained data to library entries for purified standards or unknown recurrent entities. For each sample, library matches for each compound were checked, and manually corrected if necessary. Metabolite levels were scaled in accordance with run-day medians.

### Osmolality measurements

Osmolality measurements were performed on samples submitted for metabolomics analysis in 2013 by Metabolon Inc., and on samples used with the biochemistry assay in 2015 by iTRI. Both sets of measurements were performed using a freezing-point Fiske Micro-Osmometer Model 210. Osmolality was determined using 20 µL of sample, and measurements were performed in duplicate. The osmometer was calibrated daily using standards for saliva (50 mmol/kg) [26].

### Biochemical assay to measure 1,5-AG

We attempted to quantify 1,5-AG in the saliva and plasma samples using the commercially available GlycoMark™ kit (Tomen America, New York, NY), following the manufacturer’s recommendations as previously described [16].

The assay consists of two main steps. In the first step, glucose is eliminated from the sample. In the second step, pyranose oxidase (PROD) kinetic efficiency of C-2 hydroxyl group oxidation of 1,5-AG is measured by hydrogen peroxide formation, which is detected by colorimetry using peroxidase (POD). This reaction can be monitored at 546 nm (primary wavelength). To correct for background or nonspecific reading the absorbance at 700 nm is also measured. Glucose is eliminated from the sample because it can serve as a substrate for PROD.

The kit components allow execution of these steps in a clinical chemistry analyser. Briefly, all reactions were performed at 37 °C in the RX daytona^+^ clinical chemistry analyser (Randox Laboratories, Ltd.) programmed according to the manufacturer’s protocol. The kit includes two different reagents (Reagent 1 and Reagent 2) that are optimized for two sequential steps performed in a fully automated way. First, 4 µL of sample was mixed with 120 µL of Reagent 1 and incubated for 5 min. Reagent 1 contains glucokinase to eliminate glucose from the sample. Next, 60 µL of Reagent 2, containing PROD was added, and the reaction kinetic was monitored by measuring absorbance immediately, and after 10 min at 546 nm (primary wavelength) and 700 nm (secondary wavelength).

The 1,5-AG concentration in the sample is automatically calculated as follows: (1) The absorbance values determined at 700 nm were subtracted from those determined at 546 nm; (2) The kinetic difference in absorbance between the second and first measurements was calculated; (3) The 1,5-AG concentration in the sample was determined by comparison with the kinetic changes in the absorbance of the reagent blank and of the standard provided in the kit.

To ensure reproducibility, the instrument was calibrated daily with a calibration standard provided by GlycoMark™. To verify measurement precision, we included two levels of controls prepared in surrogate serum matrix with 1,5-AG concentrations of ~4.0–5.5 µg/mL (low-level control) and ~13.0–16.0 µg/mL (high-level control), both provided by GlycoMark™.

### Statistical data analysis

All statistical analyses were performed using R version 3.1.3 and R-Studio version 0.97.551. The package ‘stats’ version 3.1.3 was used to calculate linear regression. For each identified metabolite the raw area ion counts were normalized to the median value of the run day and the data were z-scored before computing the statistics. Linear regression was applied to assess the statistical significance of the association between the metabolite intensities determined using non-targeted metabolomics approaches and the 1,5-AG concentrations determined by biochemical assay. The statistical significance of the correlation between the results of non-targeted metabolomics and the 1,5-AG measurements performed via biochemical assay was inferred with the use of stringent Bonferroni correction to account for testing 1360 metabolites (*p* < 0.05/1360 = 3.67 × 10^−5^).

## Results

### Correlation between mass spectrometry and biochemical assay measurements of 1,5-AG in blood

We first tested the compatibility of the Glycomark assay with the RX daytona^+^ clinical chemistry analyser (Randox Laboratories, Ltd.), as this equipment was not explicitly specified by the manufacturer. To verify that the assay functioned correctly on this instrument, we determined the measurement variability of the high-level (~4.0–5.5 µg/mL 1,5-AG) and low-level (~13.0–16.0 µg/mL 1,5-AG) quality controls. Over a period of 5 days, we measured a total of 42 low-level and 42 high-level quality control samples. The average measured concentrations were 4.94 and 14.57 µg/mL, respectively. Day-to-day variability in the measurements was minimal for both low-level (CV = 1.8 %) and high-level (CV = 1.38 %) quality control samples. Our measurements were comparable to previous measurements performed using the Glycomark-certified Hitachi 917 analyser (low-level control, 4.7 µg/mL, CV = 3.71 %; and high-level control, 14.7 µg/mL, CV = 1.35 % [16]), thus demonstrating that the Glycomark assay is also compatible with the presently used RX daytona^+^ clinical chemistry analyser.

**Fig. 1:**
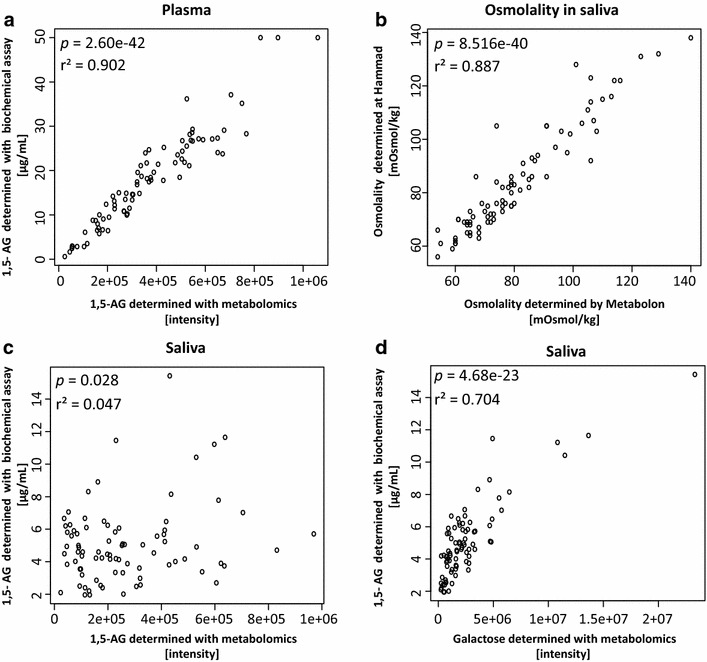
**a** Correlation between 1,5-AG intensities determined using the non-targeted semi-quantitative mass spectrometry (MS)-based metabolomics platform and the 1,5-AG concentrations measured with the quantitative Glycomark biochemical assay in two plasma aliquots from the same patient. **b** Correlation between osmolality measurements of saliva performed using identical instruments but on two individual platforms (TRI and Metabolon) with a time interval of 2 years. **c** Correlation between 1,5-AG intensities determined using the non-targeted MS-based metabolomics platform and the 1,5-AG concentrations determined with Glycomark assay in saliva. **d** *Scatterplot* of Glycomark assay read-outs from saliva and galactose intensities determined using the non-targeted MS-based metabolomics platform

Next, we tested whether the 1,5-AG concentrations determined with the Glycomark assay correlated with the semi-quantitative 1,5-AG levels measured in plasma using non-targeted metabolomics (expressed in run-day normalized ion-counts). We found a highly significant correlation between both measurements in plasma samples, as shown in Fig. 1a (*p* = 2.6 × 10^−42^, r^2^ = 0.902). This finding demonstrated the reproducibility of non-targeted metabolomics results using the biochemical assay, simultaneously confirming the high quality of both analytical methods.

We also examined whether the assay read-outs from the plasma samples correlated with any other metabolite measured using the non-targeted metabolomics platform. We found two unknown metabolites X-19437 (p value = 4.85×10^−6^, r^2^ = 0.22) and X-18221 (p-value = 1.25×10^−4^, r^2^ = 0.16), which showed significant correlation with assay readouts.

### Low variability of the Glycomark assay for saliva

Novatzke et al. [16] previously evaluated the variability of the Glycomark assay by assaying two replicates of pooled serum samples, twice a day, over a period of 10 days. The between-assay variability of pooled serum samples was very low (CV = 1.17 %) and comparable with quality controls. Here we evaluated the variability of the Glycomark assay by measuring 20 samples of pooled saliva in a single assay run (within-assay variability), and by measuring 59 samples in at least triplicate over a 5-day period (between-assay variability) (Table 2A). We found very low variability in the saliva measurements in a single assay run (1.7 %), and between different assay runs performed on different days (2.05 %). These values are comparable to the variability of the quality control measurements (1.4–1.8 %) and to previous findings with pooled serum samples [16]. Our results suggest that saliva is a stable matrix for application of the Glycomark™ assay.

**Table 2:** Assay variability in saliva samples. (A) Variability in 1,5-AG measurements with GlycoMark assay. (B) Variability in osmolality measurements with FISKO osmometer

	Within-assay variability	Between-assay variability		
	Master mix	Master mix	Control low	Control high
(A) GlycoMark assay^a^				
Number of samples	20	59	42	42
Average 1,5-AG, µg/mL	4.08	4.11	4.94	14.6
CV, %	1.70	2.05	1.80	1.38
(B) Osmolality measurements^b^				
Number of samples	20	59	–	–
Average osmolality, mOsmol/kg	59	59	–	–
CV, %	1.31	1.40	–	–

^a^Table shows that the GlycoMark assays yield reliable and reproductive measurements in saliva. The coefficient of variation (CV) is expressed in percentage and the assay read-out in µg/mL

^b^Table shows that the osmolality measurements have a very low variability. The coefficient of variation (CV) is expressed in percentage and the osmolality in mOsmol/kg

### Osmolality of saliva samples analysed on two independent platforms

We first evaluated the technical variability of osmolality measurements on our TRI platform using pooled saliva samples prior to the 1,5-AG measurements. Table 2B shows the low variation among measurements performed on the same day (CV = 1.3 %) and over the 5-day period (CV = 1.4 %). We next compared osmolality measurements performed using aliquots of the same saliva samples, but run at two sites (Metabolon and TRI) using same equipment. For this comparison, we determined the osmolality of 82 saliva samples for which the osmolality was previously measured at Metabolon. We found that the two data sets were highly correlated, as shown in Fig. 1b (*p* = 8.52 × 10^−40^, r^2^ = 0.887). These findings confirm the quality of the osmolality measurements and, in particular, exclude the possibility of accidental sample interchange.

### Glycomark assay read-out in saliva correlates with galactose intensity

Using the Glycomark assay, we analysed 82 saliva samples. As shown in Fig. 1c, we found a very low correlation between 1,5-AG intensities and assay read-outs (*p* = 0.028, r^2^ = 0.047). Thus, we further examined whether the assay read-outs from the saliva samples correlated with any other metabolite measured using the non-targeted metabolomics platform. Table 3 presents the 12 metabolites that we identified as being significantly correlated with the Glycomark assay read-out, which included 6 carbohydrates, 2 amino acids, 1 lipid, 1 peptide, and 2 unknown metabolites. By far, the strongest correlation was observed with galactose (*p* = 4.68 × 10^−23^, r^2^ = 0.704) (Fig. 1d). We next identified all correlations between galactose and other metabolites determined using non-targeted metabolomics in saliva. This analysis revealed 42 metabolites that significantly correlated with galactose, among which 10 were also significantly correlated with the Glycomark assay read-outs from saliva (Table 3).

**Table 3:** Metabolites significantly correlating with assay read-out overlap with metabolites significantly correlating with galactose

Metabolite	Biochemical class	Correlation with assay read-out		Correlation with galactose		lm(AG_Sal_H ~ met + galactose)
		r^2^	*p*	r^2^	*p*	*p*
*Galactose*	Carbohydrate	0.704	4.7 × 10^−23^	–	–	–
*N*-acetylglucosamine	Carbohydrate	0.436	9.2 × 10^−12^	0.787	7.8 × 10^−29^	2.5 × 10^−03^
*Fucose*	Carbohydrate	0.376	5.4 × 10^−10^	0.542	3.1 × 10^−15^	0.99
*Xylose*	Carbohydrate	0.318	2.5 × 10^−08^	0.506	6.1 × 10^−14^	0.47
*Mannose*	Carbohydrate	0.276	2.3 × 10^−07^	0.396	1.4 × 10^−10^	0.97
*O*-acetylserine	Amino acid	0.355	1.9 × 10^−06^	0.524	7.9 × 10^−10^	0.84
Putrescine	Amine	0.234	2.5 × 10^−06^	0.451	3.0 × 10^−12^	0.09
Ribose	Carbohydrate	0.232	2.7 × 10^−06^	0.403	9.0 × 10^−11^	0.31
1,2-propanediol	Lipid	0.200	1.5 × 10^−05^	0.279	2.0 × 10^−07^	0.89
X-14904	Unknown	0.186	3.0 × 10^−05^	NS	NS	2.2 × 10^−04^
X-18059	Unknown	0.186	3.1 × 10^−05^	NS	NS	5.9 × 10^−04^
Lysylproline	Peptide	0.185	3.3 × 10^−05^	0.413	4.5 × 10^−11^	0.02

The association trend was positive for all significantly correlating metabolites. We analysed correlation of those metabolites with galactose, which correlation with assay read-out was the strongest. No significance in correlation between metabolites and galactose is expressed as NS. We applied linear regression (lm) of 1,5-AG measured with Glycomark (AG_Sal_H) against 1,5-AG measured on non-targeted metabolomics platform (AG_Sal_M) and corrected on the covariate (met), listed in the “Metabolite name” column. Metabolites with similar chemical structure to 1,5-AG are highlighted in italics

## Discussion

In our present study, we evaluated a possible method for measuring 1,5-AG in saliva samples using a set-up compatible with clinical analytical laboratories. The 1,5-AG could be quantified in saliva samples using NMR as well as LC/MS based targeted metabolomics approach, however it implementation into the clinical setting is limited [27]. Thus, we deployed an automated biochemical assay for 1,5-AG concentrations (Glycomark kit) to analyse plasma and saliva samples for which metabolic profiles had previously been determined [23]. Our results showed a highly significant correlation between the plasma 1,5-AG levels determined by non-targeted metabolomics and those measured with the Glycomark assay, demonstrating a good agreement between the two methods. However, we found no corresponding correlation of apparent 1,5-AG levels in saliva samples.

Previous studies investigated and ruled out cross-reactivates of assay readouts in blood [15]. We confirmed that the dominant signal is from 1,5-AG (p-value = 2.6×10^−42^ and r^2^ = 0.902). Two metabolites of unknown id, presumably carbohydrates [24], show some correlation but the signal was much weaker than the 1,5-AG signal. However, the cross-reactivity in saliva samples was not tested before, and the lack of correlation in saliva could be due to the assay interference.

We detected a correlation between parallel osmolality measurements, excluding the possibility of potential sample switching. Further investigation revealed a high correlation between the kit read-out and the galactose intensities in saliva samples, suggesting that galactose might have interfered with the assay measurements. In the non-targeted metabolomics data, we found that the 1,5-AG read-outs of the biochemistry assay correlated with the following 12 saliva metabolites: galactose, *N*-acetylglucosamine, fucose, xylose, mannose, *O*-acetylserine, putrescine, ribose, 1,2-propanediol, lysylproline, and the unknown metabolites X-14904 and X-18059 (see Table 3), with the strongest correlation observed for galactose. Therefore, we hypothesize that galactose was the dominant molecule interfering with our 1,5-AG measurements.

This phenomenon could conceivably be associated with nonspecific activity of the enzyme involved in the final step of the Glycomark assay. In the sequential reactions, pyranose oxidase (PROD) catalyses oxidation of the C-2 hydroxyl group of 1,5-AG. However, PROD can also catalyse oxidation of several other carbohydrates at the 2-OH moiety of the pyranose ring structure [28]. Since glucose serves as a PROD substrate, the assay includes an initial step to enzymatically modify glucose and thus minimize interference [16]. In plasma, only glucose is present at a level that could potentially interfere with assay read-outs, with other monosaccharides detected at only very low levels [28]. We found that saliva also contains glucose, along with galactose, fucose, xylose, and mannose, which could each potentially serve as a PROD substrate and hence interfere with the assay read-outs. A previous study examined PROD affinity towards several sugar alcohols relative to 1,5-AG, and reported the following affinity ranking: xylose>glucose>galactose>sorbose>glucano-lactone>mannose [28]. Another study also showed that fucose is a PROD substrate [29]. Although we detected xylose and mannose in plasma samples, the concentrations were insufficient to interfere with the measurements, since we observed a significant correlation between 1,5-AG measured with non-targeted metabolomics and the Glycomark assay. In contrast, we detected glucose and several other monosaccharides in saliva. The lack of correlation between glucose and assay read-outs in saliva demonstrated that saliva provides a stable environment for glucokinase activity, which catalyses glucose modification.

Further analysis revealed that almost all metabolites (except the unknown metabolites X-14904 and X-18059) that significantly correlated with assay read-outs, also significantly correlated with galactose, regardless of their chemical structure. Furthermore, the correlations between metabolites and galactose concentrations were more strongly significant than the correlations between metabolites and the assay read-outs. Thus, we hypothesises that galactose is a major interference of the 1,5-AG measurements, and fucose, xylose, and mannose show significant correlations with the assay read-outs because they correlate with galactose rather than because they interfere with the measurements.

To investigate whether galactose was the main metabolite interfering with our measurement, we analysed the data using a linear model including galactose as a covariate. In this model, we tested the contribution of each metabolite that was found to significantly correlate with assay read-outs: (lm(AG_Sal_H~metabolite + galactose)). As shown in Table 3, none of the other metabolites contributed significantly. Hence, our data was totally dominated by the galactose signal. Furthermore, any potential contribution from 1,5-AG or other signals was lost in the background measurement noise. The galactose signal cannot be mathematically corrected and requires evaluation in the laboratory setting.

## Conclusions

With this study we showed feasibility and limitations of monitoring of 1,5-AG using Glycomark™ assay in saliva, as method to facilitate diabetes screening. The replicable assay readouts from saliva samples showed that saliva serve as stable matrix for biochemical assay. However, the signal was dominated by galactose, which is biochemically similar to 1,5-AG and absent in blood. In future experiments, we will attempt to improve the assay by introducing an enzymatic step to remove galactose before sample processing, which should increase the assay specificity for salivary 1,5-AG. Further optimization of the assay for 1,5-AG measurement in saliva will likely result in a versatile tool for diabetes screening and monitoring that will improve T2D diagnosis, especially in paediatric patients, and this is one of our future study goals.
